# Characterization of Non-Cholesterol Sterols in Microglia Cell Membranes Using Targeted Mass Spectrometry

**DOI:** 10.3390/cells12070974

**Published:** 2023-03-23

**Authors:** Ilijana Begcevic Brkovic, Madlen Reinicke, Soroth Chey, Ingo Bechmann, Uta Ceglarek

**Affiliations:** 1 Institute of Laboratory Medicine, Clinical Chemistry and Molecular Diagnostics, University of Leipzig Medical Center, 04103 Leipzig, Germany; 2 Institute of Anatomy, Faculty of Medicine, University of Leipzig, 04103 Leipzig, Germany

**Keywords:** sterols, targeted mass spectrometry, plasma membrane microdomains, microglia, plant sterols, cholesterol precursors

## Abstract

Background: Non-cholesterol sterols, as well as plant sterols, cross the blood–brain barrier and, thus, can be incorporated into cell membranes, affecting the cell’s inflammatory response. The aim of our work was to develop an analytical protocol for a quantitative assessment of the sterol composition within the membrane microdomains of microglia. Methods: A protocol for cell membrane isolation using OptiPrep^TM^ gradient ultracentrifugation, in combination with a targeted mass spectrometry (LC-MS/MS)-based assay, was developed and validated for the quantitative analysis of free sterols in microglia cell membranes. Results: Utilizing an established LC-MS/MS assay, cholesterol and seven non-cholesterol sterols were analyzed with a limit of detection from 0.001 to 0.05 mg/L. Applying the detergent-free isolation of SIM-A9 microglia cell membranes, cholesterol (CH), desmosterol (DE), lanosterol (LA) stigmasterol (ST), beta-sitosterol (SI) and campesterol (CA) were quantified with coefficients of variations between 6 and 29% (fractions 4–6, *n* = 5). The highest concentrations of non-CH sterols within the microglia plasma membranes were found in the microdomain region (DE>LA>SI>ST>CA), with ratios to CH ranging from 2.3 to 435 lower abundancies. Conclusion: By applying our newly developed and validated analytical protocol, we show that the non-CH sterol concentration is about 38% of the total sterol content in microglia membrane microdomains. Further investigations must clarify how changes in the non-sterol composition influence membrane fluidity and cell signaling.

## 1. Introduction

In mammals, cholesterol (CH) is an essential structural component of cell membranes that organizes and modulates membrane fluidity. Transient lateral liquid-ordered regions exist within the membrane [1]. Such membrane microdomains, so-called lipid rafts, are highly dynamic (transient) structures; are enriched in CH, glycosphingolipids and related proteins; and are involved in various functions, including signal transduction [2].

Non-CH sterols are either derived from endogenous cholesterol biosynthesis (e.g., lanosterol and desmosterol) or are exclusively derived from the diet (e.g., plant sterols (PSs) campesterol and beta-sitosterol). In circulation, both CH and non-CH sterols are incorporated into lipoproteins. Cholesterol precursors are known as markers of CH synthesis [3], while PSs are markers of CH intestinal absorption [4]. PSs were found to have CH-lowering effects, but they can also impair endothelial function and promote atherosclerosis at high blood and tissue concentrations [4,5,6]. However, recent studies still demonstrated the anti-inflammatory effects of PSs by illustrating their ability to downregulate pro-inflammatory transcription factors and signaling surface proteins [7,8].

The central nervous system is the most CH-rich organ in mammals, and all available CH is synthesized in situ. Disturbed CH metabolism is observed to affect neurological disorders and neuroinflammation [9]. Opposite to CH, PSs have been reported to cross the blood–brain barrier [10]. Animal investigations showed that PSs accumulate in the brain [11,12]. Recently, PSs were also identified in human cortex samples [13]. Incorporated into cell membranes, PSs may affect membrane fluidity, cell signaling and the sensitivity of cells to oxidative stress [14,15]. Like plant sterols, omega-3 polyunsaturated fatty acids (PUFAs) are exclusively derived from nutrition and are incorporated into cell membranes. Whereas the anti-inflammatory role of omega-3 PUFAs is known, increasing evidence has also linked PSs to anti-inflammatory and protective effects in the central nervous system [14,16,17], but the exact cellular mechanisms of their action are not well understood.

Microglia are brain-resident macrophages. Activated microglia are involved in the development of neuroinflammatory and neurodegenerative diseases [18]. Moreover, microglia lipid metabolism was found to be important for development and cellular function [19]. While microglial cholesterol efflux was already described [20], the influence of the sterol composition and PS content in cell membranes has not yet been investigated.

Since non-CH sterols have a 100–1000-fold lower concentration in the circulation than CH, and given that only free sterols are relevant in cell membranes, a sensitive analytical method is required to quantify the free forms of non-CH sterols in cell membranes. Therefore, the aim of our work was to develop a sensitive liquid chromatography–tandem mass spectrometry (LC-MS/MS)-based assay for the quantitative analysis of free sterols in cell membrane fractions and, second, to optimize the cell membrane isolation procedure. With our established analytical protocol, the sterol and PUFA compositions of microglia cell membranes wereinvestigated.

## 2. Materials and Methods

### 2.1. Chemicals and Reagents

Ultra-high-performance LC/MS-grade isopropanol and methanol were purchased from Biosolve (Valkenswaard, the Netherlands), while deionized, ultrapure water was produced in-house with a Barnstead Nanopure system (Thermo Fischer Scientific, Waltham, MA, USA). Campesterol (CA), stigmasterol (ST), 7-dehydrocholesterol (7-DHC), 4-cholestenone and internal standards (ISs)—ST-d5, LA-d6 and DE-d6—were purchased from Avanti Polar Lipids (Alabaster, AL, USA). Sitosterol (SI), lanosterol (LA) and CH were obtained from Merck, Sigma-Aldrich (Darmstadt, Germany), and brassicasterol (BR) and desmosterol (DE) were obtained from Steraloids (Newport, RI, USA). Finally, CA-d7 and SI-d7 were purchased from Medical Isotopes (Pelham, NH, USA), and CH-d7 was purchased from Cambridge Isotope Laboratories (Tewksbury, MA, USA). Isotope label positions are provided in Table S1.

### 2.2. Human Samples

Residual EDTA plasma samples from patients’ routine diagnostics at the University Hospital Leipzig were used for method development and validation purposes (ethical approval 082-10-190-42010). A method comparison was performed utilizing human samples from an observational trial of patients receiving cholesterol-lowering drugs. Written informed consent was obtained from all patients, and the study protocol was approved by the institution’s ethics committees on research on humans (EA4/178/15 and 162/15).

### 2.3. Sample Preparation

Samples were prepared according to an already established procedure for PS analysis in plasma [21]. A total of 10 µL of pooled plasma, reconstituted cell membrane fractions, calibrators, a blank and a double blank was added to a precipitation solution consisting of 490 µL of methanol/isopropanol (50/50, *v*/*v*) and ISs (final concentrations: 100 µg/L CH-d7, 50 µg/L CA-d7/SI-d7 and 20 µg/L DE-d6/LA-d6/d5-ST). The samples were vigorously vortexed for 10 s and centrifuged at 16,000× *g* for 10 min. The supernatant was then transferred to a glass autosampler vial and stored, when appropriate, at −80 °C until analysis. The blank, double blank and calibrators were of solvent-based origin (isopropanol instead of biological matrix).

### 2.4. LC-MS/MS Analysis

A targeted LC-MS/MS analysis was performed utilizing an HTS-xt PAL autosampler from CTC Analytics (Zwingen, Switzerland) and a Nexera XR HPLC system from Shimadzu (Duisburg, Germany), online coupled to a QTRAP 6500 (Sciex, Framingham, MA, USA) mass spectrometer, equipped with an IonDrive Turbo V ion spray source operating in the positive atmospheric pressure chemical ionization (APCI) mode. The autosampler was cooled at 10 °C, and 25 µL of the sample was injected into a 150–4.6 mm monolithic Chromolith^®^ High Resolution column (RP-18 endcapped, Merck, Darmstadt, Germany), heated at 40 °C. The mobile phases consisted of eluent A, methanol/water (75/25, *v*/*v*), and eluent B, 100% isopropanol. The flow rate was 1 mL/min with the following LC gradient profile: 0–7 min 40–50% B, 7–8 min increase to 100% B, 8–13.3 min 100%, 13.3–13.5 decrease to 40% B, 13.5–14.0 min 40% B. The ion source parameters were optimized and set to the following: temperature 450 °C, source gas 1 30 psi, nebulizer current 3 µA, curtain gas 40 psi and collision gas 8 psi. The resolution of Q1 and Q3 was set to unit. In addition, declustering potentials, collision energies, collision cell exit potentials and dwell times were optimized for every compound. The elution window was monitored between 6 and 13.3 min, while the remaining elutions were discarded as waste via the diverted MS valve. The MRM precursors, transitions and corresponding potentials are specified in Supplementary Table S1. Zymosterol (Avanti Polar Lipids, Alabaster, AL, USA) was assessed separately to investigate its separation from DE.

### 2.5. Method Validation

The method linearity for CH was assessed by performing a serial dilution of 2 g/L CH stock solution (in isopropanol). The linearity of other sterol compounds was assessed by preparing serial dilutions of the calibrator mix (100 mg/L in isopropanol) consisting of all standards (apart from 4-cholestenone). Linear regression curves were calculated with 1/x weighting. The level of detection (LOD) was estimated as the concentration with a signal-to-noise (S/N) ratio of 3, while the lower level of quantitation (LLOQ) was defined as the lowest concentration with the CV below 20%. External calibration was performed based on the area ratio of the calibrator and the corresponding isotopically labeled internal standard. Within-run and between-run precisions were determined at two levels, using native pooled plasma and spiked pool plasma samples. Recovery was determined based on an analysis of the pooled plasma before and after the spike with the equation [(final concentration—initial concentration)/added concentration], taking into account the dilution of the added compounds. The spiked amounts of BR, ST, LA, DE and 4-cholestenone were 0.96 mg/L, and those of CA, SI and 7-DHC were 4.8 mg/L.

### 2.6. Stability Assessment

The stability of the sterol compounds in the prepared pooled plasma samples (*n* = 3) was assessed for 12 h, after 24 and 48 h in the autosampler at 10 °C. The stability of the IS solution at −50 °C was investigated with a plasma pool sample (*n* = 1) prepared after one, two, three and six weeks, following the initial preparation (time zero, T = 0). To assess freeze and thaw (F/T) stability, once-frozen and thawed plasma pool samples (*n* = 3, T = 0) and processed samples (*n* = 3) were subjected to an additional 5 cycles of F/T. The result for every time point and F/T cycle is presented as a percentage difference of the peak area ratio to the mean baseline value (T = 0). Acceptable change limits (ACLs) were calculated based on the assay imprecision (for peak area ratio) multiplied by 2.77, as described in [22].

### 2.7. Method Comparison

The performance of the LC-MS/MS assay was compared with that of the gas chromatography-MS (GC-MS) assay by analyzing CA in human plasma samples (*n* = 20) with both methods. The details of CA measurements using GC-MS are already published and explained elsewhere [23]. Briefly, a sample solution consisting of plasma, IS (epicoprostanol), antioxidant (butylated hydroxytoluene, BHT) and water was subjected to hydrolysis in ethanolic KOH solution at 75 °C and derivatized to trimethylsilylether. An MS analysis was performed in the single ion monitoring mode (m/z 382.4). The total CA concentrations in µmol/L obtained using GC-MS were first transformed to mg/L units. Free CA concentrations were approximated from total CA concentrations by dividing by 6. This divider was determined in our LC-MS/MS experiments from the peak area ratio between the free CA and esterified CA of our plasma pooled samples (total peak area CA = free peak area CA + peak area esterified CA).

### 2.8. Cell Membrane Isolation

Mouse SIM-A9 microglia cells (ATCC^®^ CRL-3265, ATCC, Manassas, VA, USA) were grown for three days (until about 90% confluence) in Gibco Dulbecco’s Modified Eagle Medium: Nutrient Mixture F12 (DMEM F12, ATCC, Manassas, VA USA) medium containing 10% heat-inactivated fetal calf serum, 5% heat-inactivated horse serum and the antibiotics penicillin and streptomycin in a humidified incubator with 5% CO_2_ at 37 °C. The cell number on plates was counted before and after pooling to keep the starting cell number for cell membrane isolation constant (5 × 10^7^ cells). Cell membrane fractions were isolated using the OptiPrep^TM^ (Axis Shield, Dundee, Scotland) continuous gradient detergent-free method of Macdonald and Pike [24,25]. Briefly, four 150 cm^2^ plates of confluent SIM-A9 cells (5 × 10^7^ cells) were washed twice in PBS buffer (Gibco, MA, USA) and once with an isolation medium (IM) (250 mmol/L sucrose, 1 mmol/L, CaCl_2_ and 1 mmol/L MgCl_2_ and 20 mmol/L Tris-HCl (pH 7.8)). The cells were scraped into the IM, pelleted and then lysed in 1 mL of IM containing 1× protease inhibitor cocktail (Promega, Madison, WI, USA) via passage 20 times through a 24-gauge needle, and post-nuclei supernatants were obtained using low-speed centrifugation (1000× *g*, 10 min at 4 °C). The remaining pellet was again lysed and centrifuged twice to obtain post-nuclei supernatants. The three post-nuclei supernatants were then mixed and had a 30% OptiPrep^TM^ concentration following the addition of 1.5 volume of 50% OptiPrep^TM^. Cell membrane fractions were isolated via centrifugation for 20 h at 31,000 rpm using an SW-40 rotor in a Beckman ultracentrifuge. In total, 16 fractions of 0.7 mL were collected via aspiration from the meniscus, and the distribution of marker proteins was assessed using Western blotting. The total protein in each fraction was determined using a BCA™ Protein-Assay (Thermo Fisher Scientific, Waltham, MA, USA).

### 2.9. Western Blot

Cell membrane microdomains were confirmed using a Western blot analysis. A total of 150 µL of each fraction was precipitated using the methanol–isopropanol–water method [24], and the protein pellets were dissolved in 50 µL of 1x NuPAGE™ LDS Sample Buffer (Invitrogen, Carlsbad, CA, USA). A total of 20 µL of the protein solution was separated using SDS-PAGE, and gels were transferred to a PVDF membrane (Biorad, Hercules, CA, USA). The PVDF membrane was blocked via incubation with 5% nonfat powdered milk. The PVDF strips were incubated for 1 h (or overnight at 4 °C) at room temperature with a primary antibody (anti-flotillin-1 (Cell Signaling Technology Inc., Danvers, MA, USA) (1:1000), anti-Prohibitins PBH1 (Cell Signaling Technology Inc. Danvers, MA, USA) (1:5000) or anti-Calnexin (BD Biosciences, San Jose, CA, USA)(1:250)); washed; and then incubated with the horseradish peroxidase conjugated secondary antibody (Goat Anti-Rabbit IgG (H + L)-HRP, (Biorad, Hercules, CA, USA), dilution (1:3000) or Goat Anti-Mouse IgG (H + L)-HRP, (Biorad, Hercules, CA, USA), dilution (1:2000)). After washing, antibodies were detected using chemiluminescence with the West Pico PLUS Chemiluminescent-Substrat (Thermo Fischer Scientific, Waltham, MA, USA).

### 2.10. Lipid Extraction for LC-MS/MS Analysis

Lipids were extracted using the procedure described previously with modifications [24]. Briefly, to 150 µL of each fraction, 600 µL of a methanol/isopropanol solvent (50/50, *v*/*v*), 600 µL of chloroform and 600 µL of deionized water were added, and the tube was closed and vortexed for 1 min. The phases were separated via centrifugation at 15,000× *g* at 4 °C for 15 min. The bottom layer (chloroform/lipid phase) was transferred into a new tube. The solvent was evaporated under a nitrogen stream to dryness, and the pellet was resuspended with 100 µL of methanol/isopropanol (50/50, *v*/*v*). The samples were further analyzed with LC-MS/MS, following the sample preparation for sterols and PUFAs. Since the sterol sample preparation of membrane microdomain fractions was equal to plasma samples, IS suppression (with the highest analyte concentration) was compared to the IS of the corresponding calibrator concentration (based on area).

### 2.11. LC-MS/MS Analysis of PUFAs in Cell Membrane Fractions

An analysis of arachidonic acid (C20:4, n-6), eicosapentaenoic (C20:5, n-3) and docosahexaenoic acid (C22:6, n-3) was performed following the in-house developed and published LC-MS/MS method and sample preparation protocol [26], with slight modifications. Briefly, the LC system from Shimadzu (Duisburg, Germany) with implemented online SPE was coupled to a QTRAP^®^ 5500 mass spectrometer (Framingham, MA, USA) equipped with a Turbo V™ ion spray source, operating in the negative ion mode. The samples were stored in the autosampler at 10 °C. A total of 10 µL of the sample was loaded onto a Strata-X extraction column prior to chromatographic separation on a Kinetex^®^ C18 analytic column (column oven set to 35 °C) using a mobile-phase gradient, as described previously [26,27]. The samples were prepared following a previously published procedure for plasma samples, with some modifications [26]. All samples were prepared in the same way: 50 µL of reconstituted cell membrane fraction was added to 225 µL of a precipitation solution, consisting of methanol*BHT/H_2_O*ZnSO_4_ (80/20 *v*/*v*) (final concentrations of 17.8 g/L ZnSO_4_, 56 mg/L BHT) and IS (final concentrations: 250 ng/mL C20:4-d8, C20:5-d5 and C22:6-d5). The samples were vigorously vortexed for 2 min and centrifuged at 10,000× *g* for 5 min. The supernatant was then transferred to a glass autosampler vial and stored, when appropriate, at −80 °C prior to the LC-MS/MS analysis.

### 2.12. Data Analysis

Analyst software (Sciex, Framingham, MA, USA) was used for data evaluation and to generate linear regression curves, including linear equations and correlation coefficients. Concentrations are expressed as mean ± SD, if not otherwise stated. Graphs were created with GraphPad Prism (version 9.3.1, GraphPad Software, Inc., San Diego, CA, USA). A method comparison was carried out with the nonparametric Spearman correlation (GraphPad Prism), the Passing–Bablok regression and the Bland–Altman plot (MedCalcR 12.3., Ostend, Belgium). Sterol concentrations in the cell membrane fractions > LLOD were used for data analyses. For undetected peaks, a value of zero was used. A *p*-value < 0.05 was considered statistically significant.

## 3. Results

### 3.1. Method Development and Validation

#### 3.1.1. APCI-LC-MS/MS Sterol Method

Plasma and calibrator sample pretreatment for the analysis of free (non-esterified) sterols was adopted from the group´s previous work [21], which included protein precipitation with a sample to a methanolic IS–solution ratio of 1:50. Minor modifications were made to the IS solution content (methanol/isopropanol (50/50, *v*/*v*) vs. methanol alone) and composition (five ISs instead of a single IS as used previously), calibrator preparation (more calibrators/analytes included with a wider working range) and centrifuge speed (16,000× *g* vs. 11,400× *g*). For more details on the calibrators and IS preparation, see the Material and Methods and the Results (Method Validation) Sections.

During APCI ionization, typical (M + H − H_2_O)^+^ ions for sterol compounds were formed, while for 4-cholesteone, (M + H)+ ions were generated. One to two fragment ions per precursor, with the highest intensities and absence of matrix interferences, were selected for the final method (quantifier ions are specified in Supplementary Table S1). A multiple reaction monitoring (MRM) assay was developed for nine sterol compounds, i.e., BR, CA, SI, ST, LA, DE, 7-DHC, 4-cholestenone and CH. Examples of product ion spectra of all standards and internal standards can be found in Supplementary Figure S1.

For absolute quantification, the corresponding ISs were incorporated for each sterol, apart from BR and 7-DHC, for which the ISs CA-d7 and DE-d6 were selected for quantitative purposes, respectively.

By applying a monolithic high-resolution RP-18 column, the earliest eluting sterol was DE at 8.92 min, and SI was the latest at 10.25 min. Almost all sterols were efficiently separated during the 14 min LC run, including isobaric DE, zymosterol and 7-DHC (Supplementary Table S2). However, for 7-DHC, a broad peak at RT 9.49 min was monitored in the native plasma. This indicates that the separation of the isomers 7-DHC and 8-DHC is not sufficient (Supplementary Figure S2) [28]. BR and 4-cholestenone were not detectable in the native plasma. The RT of the corresponding precursor and fragment ions’ *m*/*z* can be found in Supplementary Table S2.

#### 3.1.2. Method Validation

The ion suppression effects of the ISs in the plasma was compared to the IS area of the corresponding sterol calibrator concentration. The percentage of ion suppression for all ISs was on average between 1 and 11% (Supplementary Table S3).

The linearity assessment results are presented in Table 1. Cholestenone was not included in the quantitative validation procedure since its corresponding IS (cholestenone-d5) interfered with the cholestenone signal. Therefore, cholestenone was qualitatively assessed.

Table 2 demonstrates the assay reproducibility and recovery values of 5 samples (i.e., between-day reproducibility was based on 25 measurements of 5 samples analyzed over 5 days). The within-day precision of the native pooled plasma and spiked plasma was below 9% and 6%, respectively. Similarly, the between-day CV of the native plasma measurements was lower than 10%, and a CV below 7% was observed for the spiked plasma. Recovery was in the range of ± 20%, with the lowest observed value of 85% (DE) and the highest of 114% (LA).

#### 3.1.3. Stability Assessment

All sterol compounds were stable in the prepared EDTA plasma samples stored at 10 °C over the course of 48 h, as demonstrated in Supplementary Figure S3. Likewise, the investigated sterols showed stability under five F/T cycles of the native plasma, as well as of the processed samples (Supplementary Figure S4). Finally, the IS solution was stable for up to six weeks of storage at −50 °C (Supplementary Figure S5). Overall, all percentage differences from the baseline were within the defined ACLs, apart from SI for F/T cycles 2 and 3 (slightly below ACL) and for the IS stability assessment at week 2 (slightly above ACL). However, no trend was observed in changed differences with further F/T cycles and IS storage duration, confirming the stability of SI.

#### 3.1.4. Method Comparison

Data on the CA method comparison between GC-MS (total sterol concentration) and LC-MS/MS (free sterols) are shown in Supplementary Figure S6. Out of 20 samples analyzed, 1 sample was assigned as an outlier, and the statistical analysis was performed on a total of 19 samples. The Spearman correlation coefficient between total CA (by GC-MS) and free CA (by LC-MS/MS) was 0.891 (*p* < 0.0001) (Figure S6a). The Passing–Bablok regression equation of the estimated free CA levels was y = 1.1363x − 0.1568, with a 98% confidence interval (CI) of the slope from 0.9480 to 1.4564 and an intercept from −0.3728 to −0.001466 (Figure S6b). On average, there was a 4.6% difference in the free CA concentration between the two methods (Figure S6c).

#### 3.1.5. Optimization of Cell Membrane Isolation for Sterol Analysis

For cell membrane isolation preparation from the SIM-A9 cells, we applied a previously described detergent-free method using OptiPrep^TM^ continuous gradient ultracentrifugation [24,25]. In total, 16 fractions were collected. We modified a previously described lipid extraction protocol for preparing the membrane fractions for our sterol LC-MS/MS analysis [24,25]. The cell number adequate for the sterol analysis from the microglia membrane was optimized to about 5 × 10^7^. In our modified protocol, the collected post-nuclear supernatants were directly processed for microdomain membrane isolation with the OptiPrep^TM^ gradient, omitting the pre-isolation step of the plasma membranes. The OptiPrep^TM^ gradient was extended to 30% (10–30%), while the centrifugation time was 20 h instead of 90 min [25]. Due to the high detection capability of the sterol LC-MS/MS method, we could apply the plasma preparation protocol to the isolated membrane fractions. The signal suppression of the ISs was on average between 2 and 4% (Supplementary Table S3).

### 3.2. Sterol Composition of Microglia Cell Membranes

**Figure 1:**
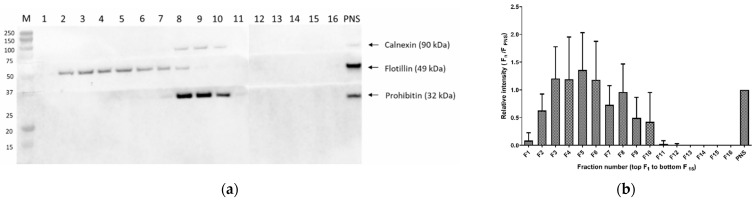

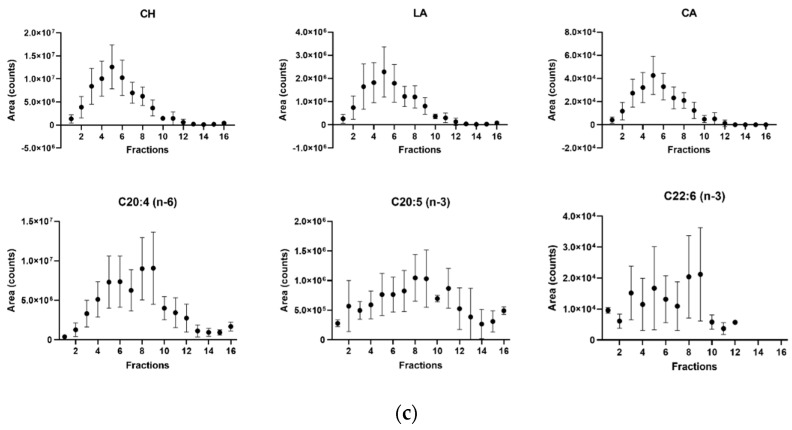
Cell membrane fractions were analyzed and evaluated using Western blot and LC-MS/MS. (**a**) Representative Western blot analysis of membrane microdomain fractions (flotillin − 1), and mitochondrial and endoplasmic reticulum (prohibitin 1 and calnexin markers, respectively) membranes in all isolated fractions (*n* = 16), obtained after ultracentrifugation of murine SIM−A9 microglia; (**b**) relative band intensity of flotillin−1 (using Western blot, *n* = 5); (**c**) LC-MS/MS analysis based on peak areas of cholesterol (CH), lanosterol (LA), campesterol (CA) and polyunsaturated fatty acids (ARA C20:4 (n − 6), EPA C20:5 (n − 3) and DHA C22:6 (n − 3)) in five consecutive isolations. Data are expressed as mean ± SD of five consecutive isolations. PNS: post-nuclear supernatant; ARA: arachidonic acid; EPA: eicosapentaenoic acid; DHA: docosahexaenoic acid.

CH, DE, LA and PSs (ST, SI and CA) were typically detectable in cell membrane fractions 2 to 11, isolated from SIM-A9 microglia. The abundancy followed the distribution of the lipid raft marker protein flotillin-1 (Figure 1c). The observed distribution based on area is equal to the area ratio distribution and absolute concentration (see Figure S7). Positive flotillin-1 detection was observed in the first seven fractions, and the non-lipid-raft markers prohibitin PBH1 and calnexin were located in the upper fractions (Figure 1a). The highest abundance of the flotillin-1 marker and, therefore, the highest abundance of the membrane microdomains were observed for fractions 4–6 (Figure 1b). The variability of the sterol distribution in five different isolations from the SIM-A9 cells was compared in the second step. In all experiments, we observed the same distribution of the protein markers and sterols in the 16 ultracentrifugation fractions, as presented in Figure 1b,c. However, the isolation CV was between 17 and 72% for fractions 2–7 when the absolute sterol concentrations were considered (see details in Section 3.3), while fractions 4–6 had a CV below 58% (Supplementary Table S4). In contrast, the isolation CV of the normalized levels to CH was below 29% (6–29%) for the same fractions 4–6. Supplementary Figure S7 demonstrates a comparison of CA concentration (mg/L) with its normalized levels and CH concentration in the microdomain fraction (2–7) over five subsequent isolations. While the concentration levels had a peak-like pattern, as previously established, the normalized values were uniform across the fractions and isolation, with a mean amount of 2.3 µg CA/mg CH. Among the normalized levels, one outlier was observed in cell membrane fraction 2 (isolation 1) (11.7 µg CA/mg CH).

In the third step, we compared the sterol distribution in the 16 cell membrane fractions with the distributions of ARA, EPA and DHA. While all sterols strictly followed the flotillin-1 abundance distribution, the PUFAs showed distinct patterns. The highest peak area was observed for fractions 8 and 9, corresponding to the Western blot band intensity of the mitochondrial protein marker prohibitin (Figure 1c).

### 3.3. Sterol Quantification in Cell Membrane Fractions and Its Relation to Plasma

Cell membrane fractions 2–7 were quantitatively evaluated to estimate sterol content. An extension of the calibration ranges from 0.05 to 50 mg/L was necessary for LA and DE (Supplementary Table S5). Representative chromatograms of the sterols and their internal standards in fraction 5 can be found in Supplementary Figure S8. Among the sterols, the highest mean concentrations were observed for CH, DE and LA, followed by SI, ST and CA, as presented in Table 3. Free SI and CA in the cell media (DMEM F12 supplemented with 10% fetal calf serum and 5% horse serum) had concentrations of 0.045 mg/L and 0.020 mg/L, respectively, while ST was below the LOD.

The absolute concentration of CH precursor DE was only 2.4-fold lower than the CH concentration in all microdomain membrane fractions, whereas LA had a 5-fold lower concentration. On the contrary, PSs were only 0.7% of the CH content. In contrast, in the plasma, all non-cholesterol sterols had a more than 1000-fold lower concentration than CH.

Interestingly, the ratios of DE and LA to CH levels were considerably higher in the membrane microdomain region than in the plasma, encompassing about three orders of magnitude (1000 and 700 times higher, respectively). While the SI/CA-to-CH ratio in the membrane microdomain region was approximately in the same range as the one in the plasma, the ratio ST/CH was around 10 times higher.

## 4. Discussion

The here presented analytical method requires a small sample volume (10 µL), simple preparation and a run time of only 14 min for the LC-MS/MS analysis of nine sterols, including isobaric components and six ISs. The method was designed for the quantification of free sterols in cell membrane fractions with sufficient sensitivity without the need for saponification or derivatization. In microglia SIM-A9 cell membranes, the cholesterol precursors DE and LA and the PSs SI, ST and CA (in descending abundancy) could be quantified reproducibly with the highest abundancy in the microdomain fractions.

The role of lipid intake in brain physiology and the development of neurodegenerative diseases is not well understood. Disturbed CH metabolism in the brain is related to the development of neuroinflammation and neurodegenerative diseases [29]. Cholesterol homeostasis is essential for proper cell membrane integrity and function. Similar to CH, non-CH sterols can be incorporated within membrane microdomains, since they share similar structural and functional properties. Due to the fact that non-CH sterols have 100–1000-fold lower concentrations in the circulation than CH, and given that only free sterols are relevant in plasma membranes, a sensitive analytical method is required to quantify the free forms of non-CH sterols in cell membranes. There are several published quantitative sterol LC-MS/MS methods with application in various biological specimens using different ionization sources [21,30,31,32,33]. Specifically, the methods developed for human plasma/serum vary in several aspects, such as the sample volume required, sample preparation procedures, the duration of the LC gradient and the number of sterols encompassed. For the determination of sterols in cell membrane fractions, Shi et al. published an HPLC method in 2013. In comparison to the described methods [30,32,33], including our previously published method [21], our developed method is based on a simple protein precipitation in combination with LC-MS/MS for the quantification of nine free sterols, including CH precursors and PSs, in plasma and cell membranes. The limits of detections were estimated to be between 0.01 and 0.05 mg/L. Using our modified detergent-free protocol for cell membrane isolation, we could show for SIM-A9 microglia cells that the total variability of both the in vitro model and the analytical protocol for the determination of sterol concentrations in the membrane microdomain fractions 4–6 ranged between 6 and 29% (normalized by CH content). The obtained variability of 29% appears to be high for LC-MS/MS analytics, but the complexity of the whole experimental procedure (cell growth on multiple plates, cell membrane isolation, sterol sample preparation and LC-MS/MS analysis) has to be considered. It still underlies the reproducibility of our results and allows us in the future to study the change in sterol composition in the cell membrane (upon different stimuli) for investigation of its functional relevance.

Once taken up by cells, sterols are, similarly to PUFAs, incorporated into membranes. We chose for our investigations the microglial cell line SIM-A9 as a model system for cell membrane characterization, because in vitro microglia activation triggers pro-inflammatory responses [19]. After cell membrane isolation, we could detect using our LC-MS/MS assay the sterols DE and LA and the PSs SI, ST and CA typically in cell membrane fractions 2–11. Non-CH sterols showed the same distributions as CH, with the highest abundancy in fractions 5 and 6. The same distribution was found for flotillin-1, indicating an enrichment of non-CH sterols in the microdomain region. It is known that, in cell membranes, CH forms together with sphingolipid microdomains, thus playing an important role in the modulation of membrane trafficking and signal transduction [34]. In contrast to sterols, the PUFAs ARA, EPA and DHA showed another distribution with the highest abundance in mitochondrial membranes. PUFAs are components of membrane phospholipids, from where they can be released by phospholipase A2 (PLA2), resulting in the formation of lipid mediators [35]. The role of omega-6 and omega-3 PUFAs in mediating inflammatory or anti-inflammatory effects on microglia activation and neuroinflammation is currently under discussion [36,37]. Less is known about the role of non-CH sterols. In previous studies, SI was identified in the plasma membranes of hippocampal neuronal cells and macrophages [7,14,16]. In addition, CA was assessed in the membrane microdomains of murine tissue [10]. However, no data are available in the literature for the CH precursors DE and LA.

In our study, we quantified the non-cholesterol sterols DE and LA and the plant sterols SI, ST and CA in mean concentration ratios to CH of 0.44, 0.20, 0.004, 0.003 and 0.002 in quantified membrane microdomain fractions 2–7. The CH precursors DE and LA had only 2.3- and 5-fold lower concentrations than CH in the membrane microdomains. In contrast, the ratio of the CH precursors to CH in the plasma is 1:100–1:1000 [3,21]. PSs are suspected of altering membrane signaling by reducing the molecular order in the membrane microdomain region [10]. We found that PSs have about 300-fold lower concentrations than CH in cell membrane fractions with the highest abundancy in fractions 4 to 6. This is in accordance with previous findings [10]. However, for ST, the ratio to CH in the cell membrane fractions was 10-fold higher than that in the plasma.

In conclusion, this study demonstrates the successful development, validation and implementation of the quantitative, multiplex LC-MS/MS assay and isolation protocol for sterol analyses in cell membrane microdomains. The method has wide applications, e.g., in human plasma and membranes, but also in other body fluids or tissues, requiring the use of a simple preparation protocol and small sample volumes (10 µL). Future work will be focused on revealing the functional importance of sterol composition in cell membrane microdomains.

## Figures and Tables

**Table 1:** Linearity and calibration range.

Sterol	LOD (mg/L)	LLOQ (mg/L)	Linear Range (mg/L)	R^2^	Calibration Range (mg/L)	R^2^
Brassicasterol	0.05	0.1	0.1–10	0.9984	0.1–10	0.9984
Campesterol	0.005	0.05	0.05–10	0.9986	0.1–10	0.9986
Stigmasterol	0.05	0.1	0.1–10	0.9988	0.1–10	0.9988
Sitosterol	0.005	0.05	0.05–10	0.9990	0.1–10	0.9990
Lanosterol	0.005	0.05	0.05–10	0.9984	0.1–10	0.9984
Desmosterol	0.01	0.05	0.05–10	0.9988	0.1–10	0.9986
7-Dehydrocholesterol	0.05	0.05	0.05–10	0.9982	0.1–10	0.9980
Cholesterol	0.0125	2.5	2.5–1000	0.9992	10–1,000	0.9992

**Table 2:** Reproducibility and recovery assessment (*n* = 5) in native and spiked EDTA plasma samples.

Sterol Compound	Concentration (mg/L)		Within-Day CV (%) ^3^		Between-Day ^4^ CV (%)		Recovery (%)
	Native	Spiked	Native	Spiked	Native	Spiked	
Brassicasterol	NA	1.16 ± 0.061	NA	5.4	NA	5.2	NA
Campesterol	0.723 ± 0.031 ^1^	5.48 ± 0.292	4.0	5.7	4.2	5.3	97
Stigmasterol	0.147 ± 0.014	0.991 ± 0.053	8.9	5.0	9.6	5.4	87
Sitosterol	0.680 ± 0.021	5.82 ± 0.360	3.1	5.8	3.1	6.2	94
Lanosterol	0.130 ± 0.004	1.226 ± 0.053	3.2	4.1	3.3	4.3	114
Desmosterol	0.167 ± 0.011	0.960 ± 0.043	5.7	4.7	6.6	4.5	85
7-Dehydrocholesterol	0.470 ± 0.034	5.64 ± 0.306	3.8	4.7	7.2	5.4	99
Cholesterol	509 ± 12.3	NA	2.6	NA	2.5	3.5	NA
4-cholestenone	NA	1.33 ± 0.073 ^2^	NA	4.2	NA	5.5	NA

^1^ Mean ± SD; ^2^ expressed as area ratio; ^3^ mean within-day CV; ^4^ *n* = 5 analyzed over 5 days; NA, not available.

**Table 3:** Concentration of the identified free sterols in plasma membrane microdomain fractions LF2-LF7 and plasma. Mean ratios of non-cholesterol sterol to CH in cell membrane microdomains and plasma.

	Sterol Compound					
mg/L	Cholesterol	Lanosterol	Desmosterol	Sitosterol	Campesterol	Stigmasterol
LF2 *	17.1 ± 11.5 (30.2)	3.7 ± 2.6 (6.9)	7.3 ± 4.3 (11.2)	0.070 ± 0.032 (0.085)	0.041 ± 0.017 (0.044)	0.057 ± 0.033 (0.080)
LF3	38.4 ± 20.7 (54.1)	7.8 ± 4.8 (12.6)	15.7 ± 7.6 (18.5)	0.120 ± 0.057 (0.149)	0.070 ± 0.031 (0.080)	0.100 ± 0.031 (0.086)
LF4	44.1 ± 18.4 (42.9)	8.6 ± 4.8 (11.6)	18.0 ± 6.4 (13.4)	0.135 ± 0.050 (0.111)	0.079 ± 0.030 (0.063)	0.111 ± 0.024 (0.066)
LF5	55.4 ± 20.8 (49.2)	11.0 ± 6.0 (15.4)	22.9 ± 7.3 (16.0)	0.175 ± 0.060 (0.143)	0.098 ± 0.031 (0.075)	0.137 ± 0.023 (0.058)
LF6	45.0 ± 17.7 (45.5)	9.1 ± 5.2 (13.2)	19.1 ± 6.3 (14.8)	0.143 ± 0.050 (0.127)	0.084 ± 0.028 (0.071)	0.133 ± 0.022 (0.052)
LF7	31.0 ± 9.6 (25.4)	5.9 ± 1.9 (4.9)	13.2 ± 4.3 (10.7)	0.105 ± 0.031 (0.077)	0.062 ± 0.014 (0.037)	0.098 ± 0.037 (0.096)
Plasma	471.7 ± 35.0 (62)	0.137 ± 0.006 (0.010)	0.177 ± 0.011 (0.022)	0.632 ± 0.048 (0.092)	0.642 ± 0.015 (0.030)	0.164 ± 0.0032 (0.006)
(FR2-7/CH) × 1000	NA	200 ± 45 (130)	440 ± 72 (340)	3.8 ± 2.3 (13)	2.3 ± 1.8 (10.1)	2.9 ± 1.2 (6.5)
(Sterol/CH) × 1000 plasma	NA	0.29 ± 0.016 (0.020)	0.38 ± 0.021 (0.040)	1.3 ± 0.030 (0.060)	1.4 ± 0.08 (0.15)	0.35 ± 0.026 (0.050)

* Concentrations expressed as mean ± SD (range). LF, lipid raft fraction; LR, lipid raft.

## Data Availability

The data presented in this study are available in the article and in the Supplementary Materials Section. Any other details are available on request from the corresponding author.

## Supplementary Materials

The following supporting information can be downloaded at https://www.mdpi.com/article/10.3390/cells12070974/s1, Table S1: Sterol compounds and MS method parameters; Table S2: Retention time of corresponding precursor and fragment ions; Table S3: Internal standard ion suppression in plasma and cell membrane fractions; Table S4: Variability of sterol levels between five cell membrane isolations; Table S5: Calibration range of quantified sterols in cell membranes; Figure S1: Product ion spectra of standards and internal standards; Figure S2: Representative chromatograms of DE and 7-DHC; Figure S3: Stability of sterols in EDTA plasma at 10 °C over 48 h; Figure S4: Stability of sterols in EDTA plasma (native and processed) during repeated freeze and thaw cycles; Figure S5: Internal standard stability stored at −50 °C over time; Figure S6: Method comparison of the established sterol LC-MS/MS assay with the GS-MS assay; Figure S7: Campesterol quantitative abundance in membrane microdomain fractions; Figure S8: Representative chromatograms of sterols in fraction 5.
