# Supplementary material for: Characterization of Non-Cholesterol Sterols in Microglia Cell Membranes Using Targeted Mass Spectrometry
Source: Cells. 2023 Mar 23;12(7):974. doi: 10.3390/cells12070974 (PMC10093698; doi:10.3390/cells12070974)

Appendix

Supplementary Material

Figure S1. Product Ion Spectra of standards and internal standards.

Standards

Brassicasterol

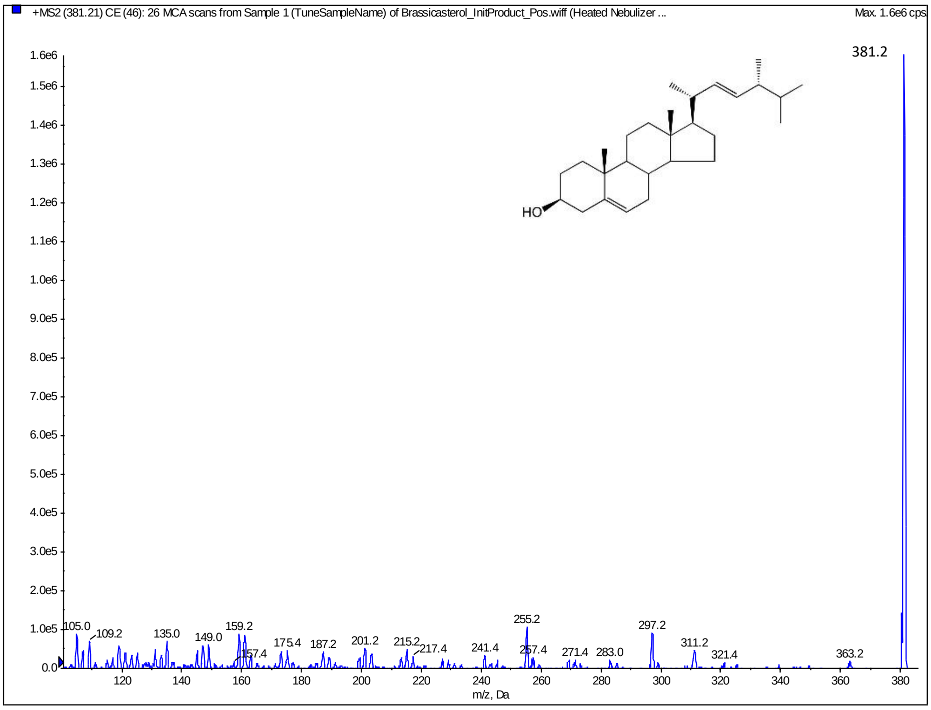

Campesterol

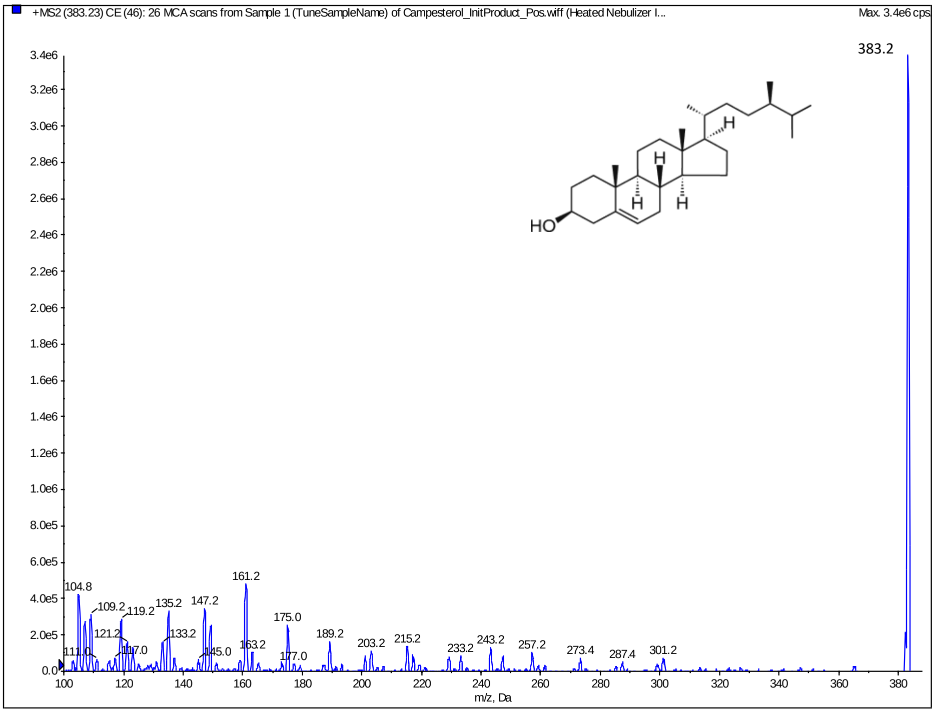

Stigmasterol

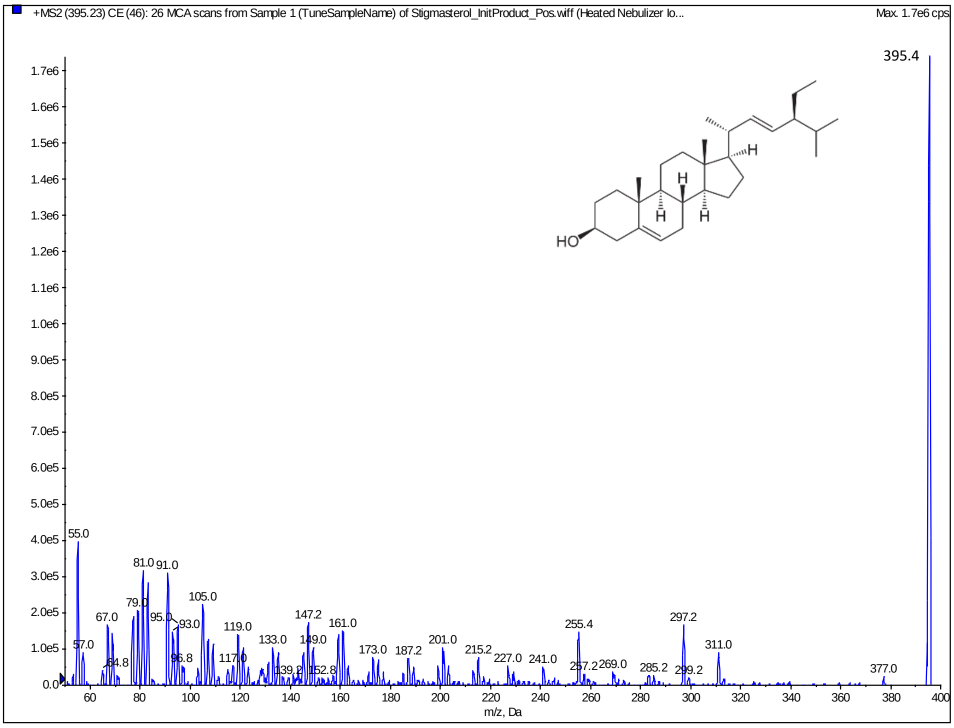

Sitosterol

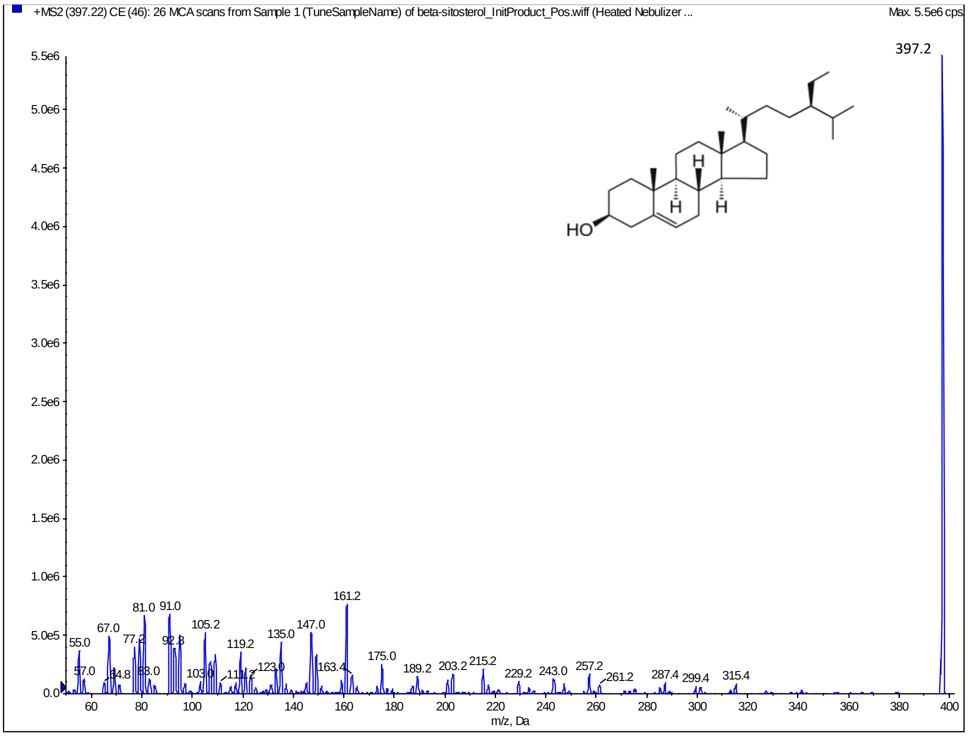

Lanosterol

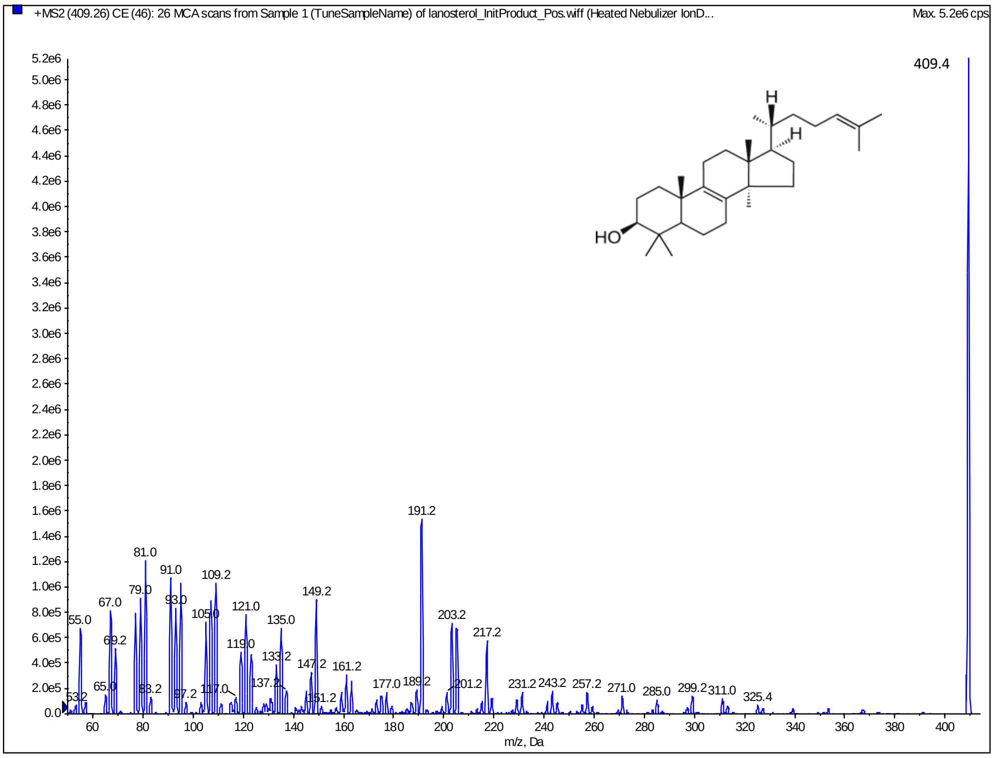

Desmosterol

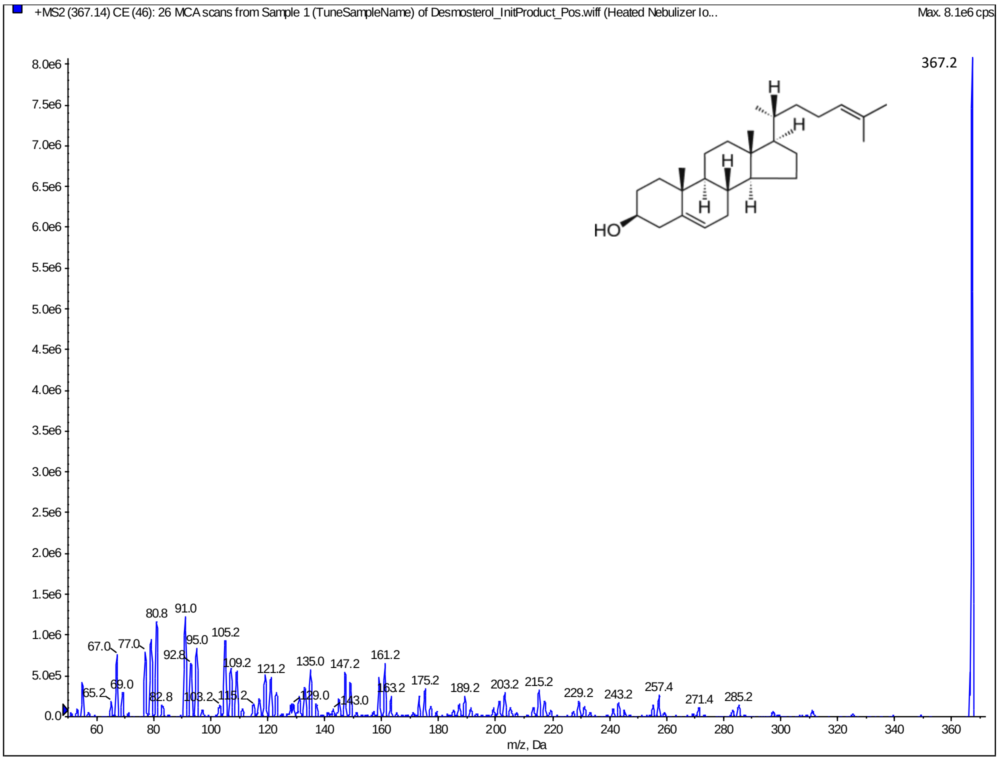

Cholesterol

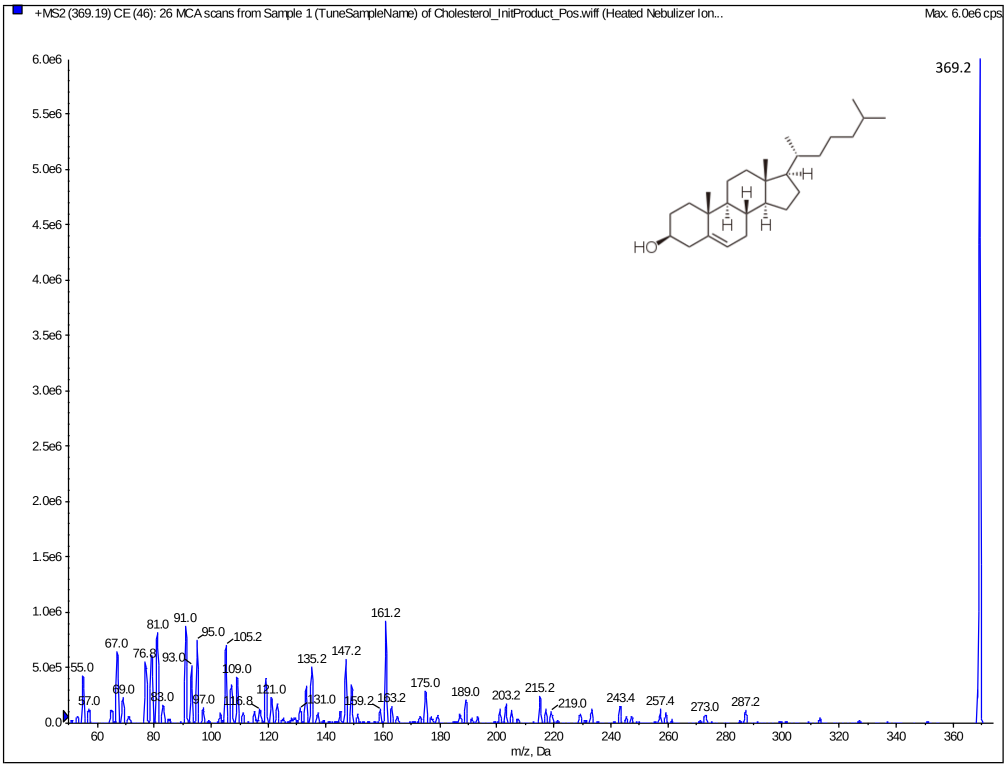

7-dehydrocholesterol

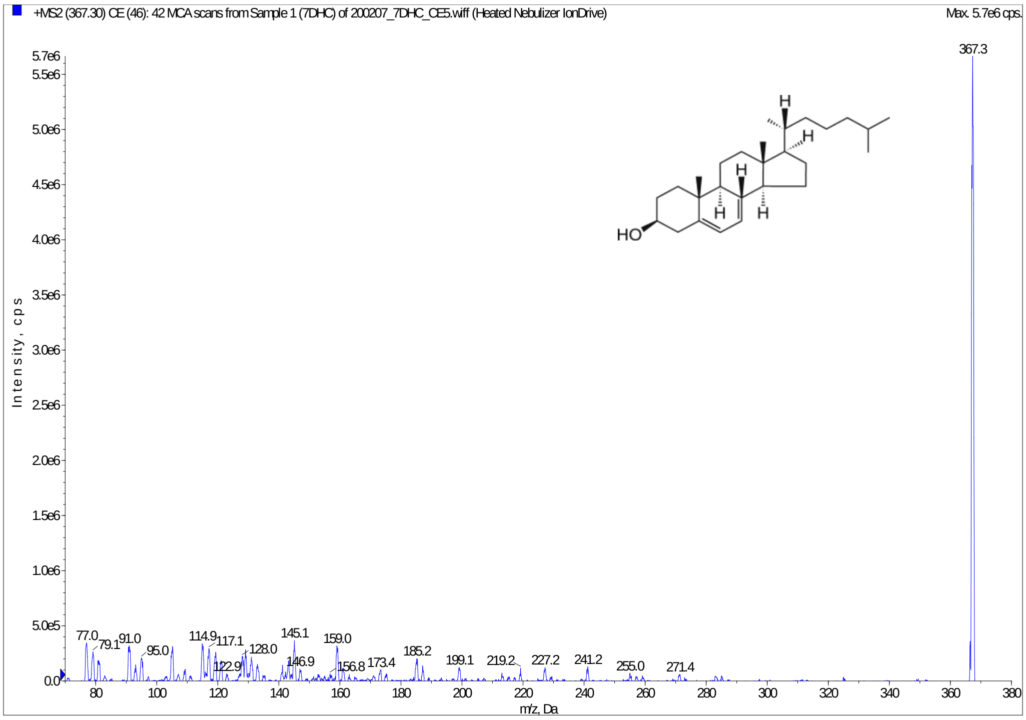

4-cholestenone

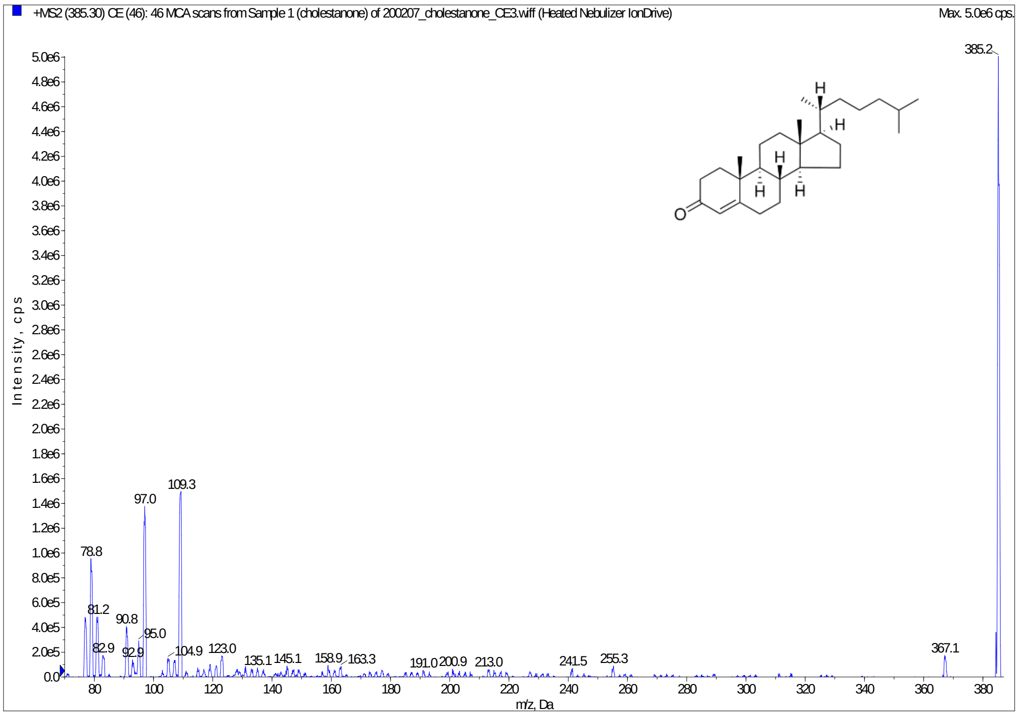

Internal Standards

d7-Campesterol

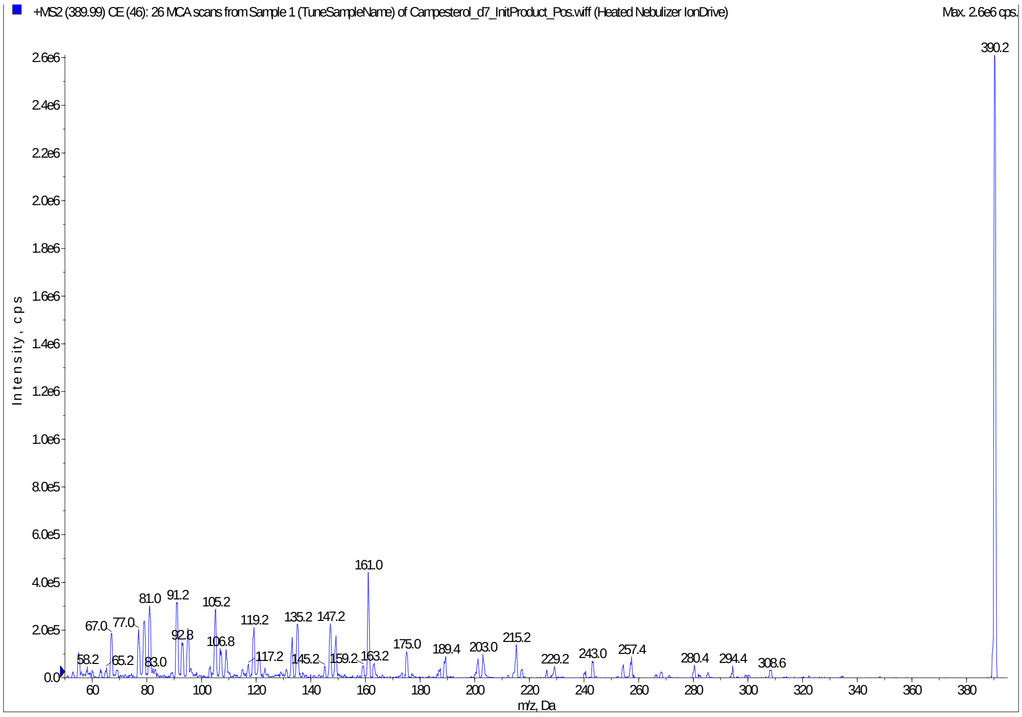

d7-Sitosterol

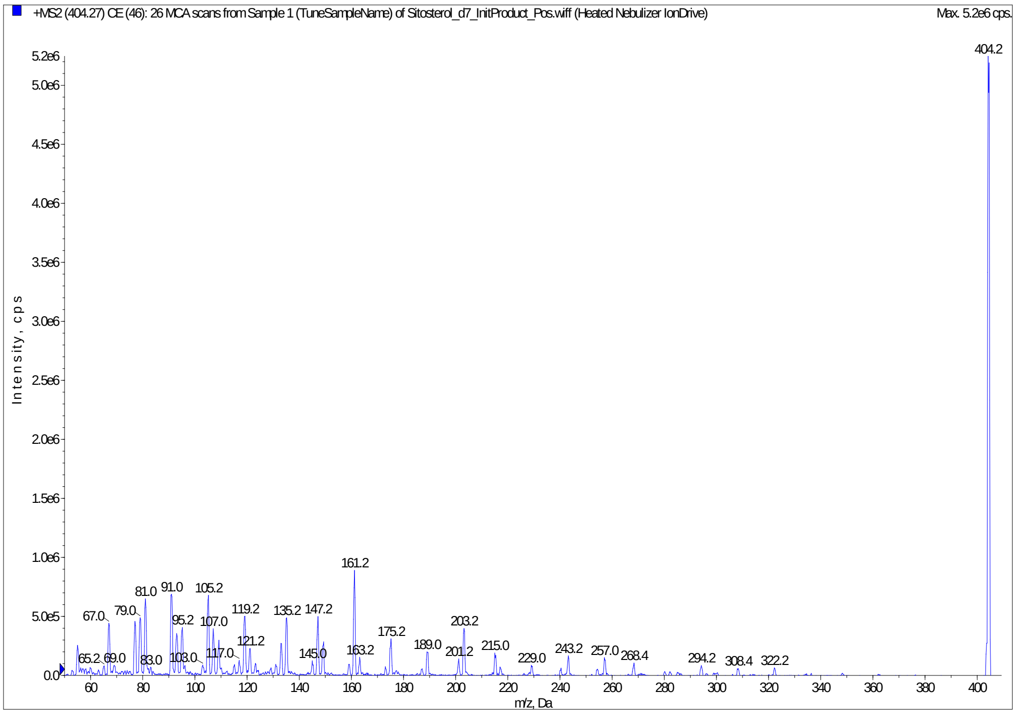

d7-Cholesterol

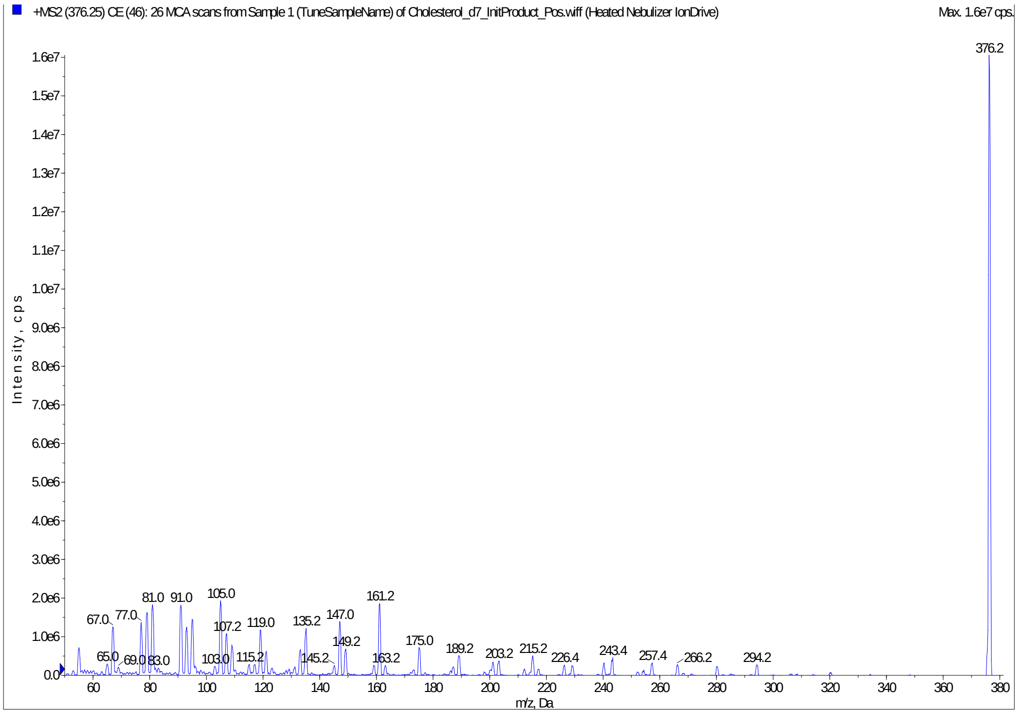

d5-Stigmasterol

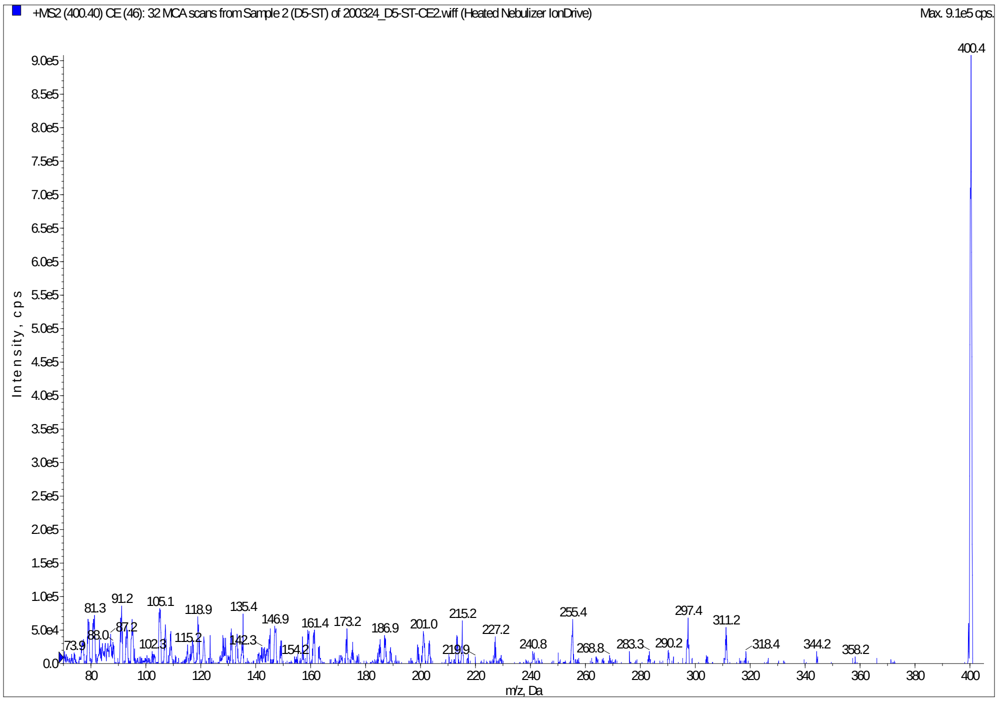

d6-Desmosterol

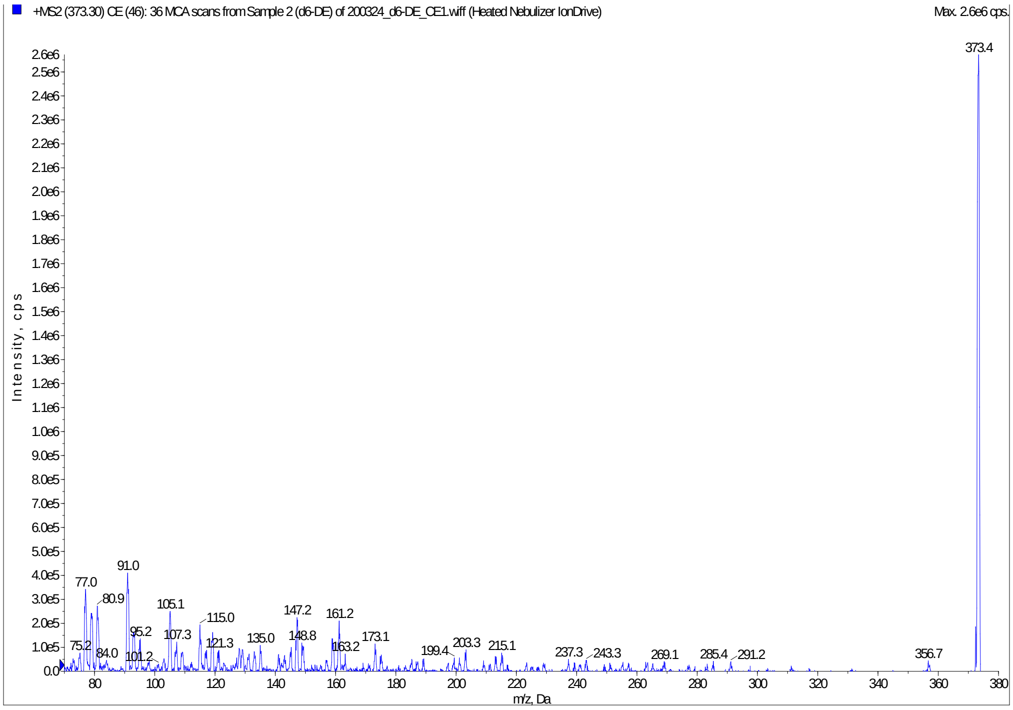

d6-Lanosterol

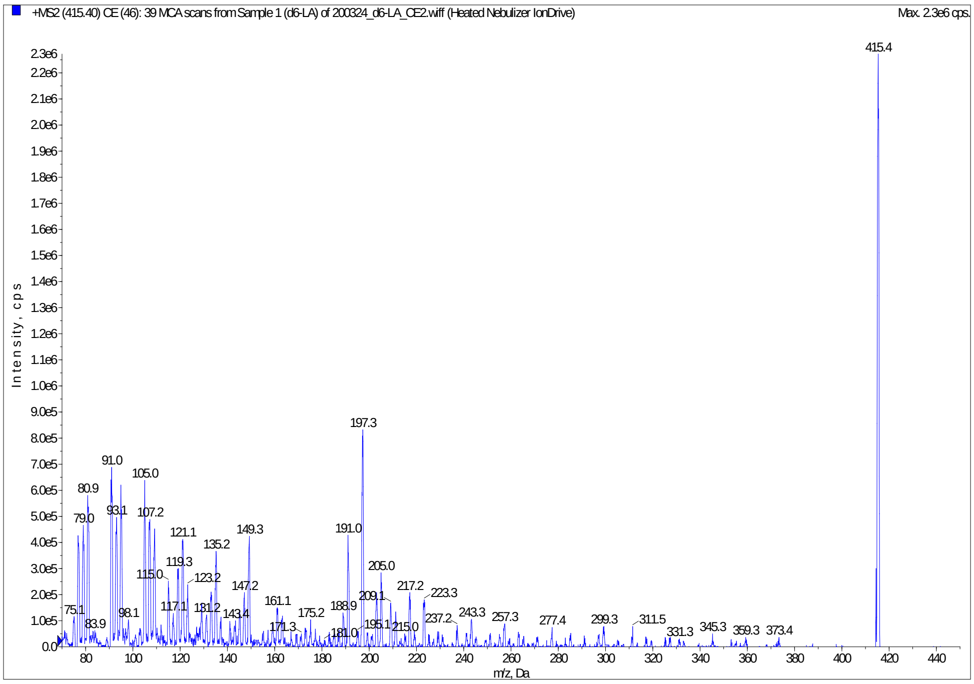

Supplement: Supplementary file 1 [file cells-12-00974-s001.zip › cells-2120603-figure s1.pdf]
