# Supplementary material for: Characterization of Non-Cholesterol Sterols in Microglia Cell Membranes Using Targeted Mass Spectrometry
Source: Cells. 2023 Mar 23;12(7):974. doi: 10.3390/cells12070974 (PMC10093698; doi:10.3390/cells12070974)
Supplement: Supplementary file 1 [file cells-12-00974-s001.zip › cells-2120603-supplementary.pdf]

Appendix

Supplementary Material

Article

Characterization of Non-Cholesterol Sterols in Microglia Cell Membranes Using Targeted Mass Spectrometry

Ilijana Begcevic Brkovic, Madlen Reinicke, Soroth Chey, Ingo Bechmann and Uta Ceglarek

Table S1. Sterol compounds and MS method parameters.

Table S2. Retention time of corresponding precursor and fragment ions.

Table S3. Internal standard ion suppression in plasma and cell membrane fractions.

Table S4 Variability of sterol levels between five cell membrane isolations.

Table S5. Calibration range of quantified sterols in cell membranes.

Figure S2. Representative chromatograms of DE and 7-DHC.

Figure S3. Stability of sterols in EDTA-plasma at 10 °C over 48 hours.

Figure S4. Stability of sterols in EDTA-plasma (native and processed) during repeated freeze and thaw cycles.

Figure S5. Internal standard stability stored at -50 °C over time.

Figure S6. Method comparison of the established sterol LC-MS/MS assay with the GC-MS assay.

Figure S7. Campesterol quantitative abundance in membrane microdomain fractions.

Figure S8. Representative chromatograms of sterols in fraction 5.

Table S1. Sterol compounds and MS method parameters.

Sterol compound	Precursor (m/z)	Fragment (m/z)	Dwell time (ms)	DP (V)	CE (V)	CXP (V)
Brassicasterol	381.3	297.1*	30	30	23	15
Campesterol	383.3	161.2*	20	50	31	9
		135.1			28	10
Stigmasterol	395.3	297.1*	30	30	27	12
Sitosterol	397.4	161.2*	10	40	31	10
Lanosterol	409.3	217.3	30	60	24	16
		191.1*			19	15
Desmosterol	367.4	161.2*	20	40	26	10
Cholesterol	369.3	105.0*	10	60	61	30
7-Dehydrocholesterol	367.3	145.1	20	54	38	20
		91.0*			86	18
4-Cholestenone	385.3	109.3	10	60	35	14
		97.0*			28	10
**Internal standards**						
d7-Campesterol	390.4	161.2	20	50	27	23
d7-Sitosterol	404.4	161.1	10	35	32	20
d7-Cholesterol	376.4	147.1	10	50	29	15
d5-Stigmasterol	404.4	297.3	20	46	23	10
d6-Desmosterol	373.3	161.3	10	27	32	8
d6-Lanosterol	415.4	91.0	20	51	89	11

*quantifier

DP- declustering potential; CE- collision energy; CXP- collision cell exit potential. Isotope label positions: ST-d5 28-D_2, 29-D_3; LA-d6 and DE-d6 26-D_3, 27-D_3; CA-d7, SI-d7 and CH-d7 25-D_1, 26-D_3, 27-D_3.

Table S2. Retention time of corresponding precursor and fragment ions.

Sterol compound	Precursor (m/z)	Fragment (m/z)	RT
Zymosterol*	367.4	161.2	8.63
Desmosterol	367.4	161.2	8.92
7-Dehydrocholesterol	367.3	91.0	9.59
Brassicasterol	381.3	297.1	9.87
4-Cholestenone	385.3	97	9.88
Cholesterol	369.3	105.0	10.00
Lanosterol	409.3	191.1	10.08
Stigmasterol	395.3	297.1	10.12
Campesterol	383.3	161.2	10.15
Sitosterol	397.4	161.2	10.25
**Internal standards**			
d6-Desmosterol	373.3	161.3	8.84
d7-Cholesterol	376.4	147.1	9.97
d6-Lanosterol	415.4	91.0	10.06
d5-Stigmasterol	404.4	297.3	10.11
d7-Campesterol	390.4	161.2	10.13
d7-Sitosterol	404.4	161.1	10.24

*not part of the method, tested for efficient separation from DE

Table S3. Internal standard ion suppression in plasma and cell membrane fractions.

Internal standard	Area* plasma (counts)	Area* calibrator (counts)	IS suppression (%)
d7-Campesterol	$1.407x10^6$	$1.580\ x10^6$	11.0
d7-Sitosterol	$1.193\ x10^6$	$1.347\ x10^6$	11.0
d7-Cholesterol	$1.530\ x10^6$	$1.505\ x10^6$	2.0
d5-Stigmasterol	$1.113\ x10^5$	$1.247\ x10^5$	11.0
d6-Desmosterol	$5.390\ x10^5$	$5.330\ x10^5$	1.0
d6-Lanosterol	$4.070\ x10^5$	$4.52\ x10^5$	10.0
**Internal standard**	**Area# LR (counts)**	**Area* calibrator (counts)**	**IS suppression (%)**
d7-Campesterol	$1.516\ x10^6$	$1.580\ x10^6$	4.0
d7-Sitosterol	$1.282\ x10^6$	$1.333\ x10^6$	4.0
d7-Cholesterol	$1.908\ x10^6$	$1.870\ x10^6$	2.0
d5-Stigmasterol	$1.180\ x10^5$	$1.247\ x10^5$	5.0
d6-Desmosterol	$5.182\ x10^5$	$5.377\ x10^5$	4.0
d6-Lanosterol	$4.438\ x10^5$	$4.577\ x10^5$	3.0

* expressed as mean, n=2-3

# expressed as mean, n=5. LR- lipid raft fraction with the highest analyte concentration

Table S4. Variability of sterol levels between five cell membrane isolations.

Isolation CV (conc.), n=5	Cholesterol	Lanosterol	Desmosterol	Sitosterol	Campesterol	Stigmasterol
**CV (LF2), %**	67.4	72.1	58.6	46.0	40.3	58.1
**CV (LF3), %**	53.8	62.3	48.3	47.2	44.3	30.6
**CV (LF4), %**	41.8	55.2	35.5	33.5	33.2	22.0
**CV (LF5), %**	37.5	54.4	31.9	33.9	31.8	17.2
**CV (LF6), %**	39.4	57.7	33.2	34.9	33.0	19.5
**CV (LF7), %**	31.0	32.0	32.6	29.5	22.4	38.1
**Isolation CV (sterols/CH), n=5**						
**CV (LF2), %**	NA	17.4	27.2	83.1	99.2	66.0
**CV (LF3), %**	NA	21.9	15.0	22.9	36.2	55.8
**CV (LF4), %**	NA	23.9	13.5	11.9	11.7	28.7
**CV (LF5), %**	NA	24.1	12.6	5.6	7.3	26.0
**CV (LF6), %**	NA	29.1	10.1	9.0	10.4	22.7
**CV (LF7), %**	NA	29.1	12.4	11.4	11.7	25.0

LF- lipid rafts fraction; CH- cholesterol

Table S5. Calibration range of quantified sterols in cell membranes.

Sterol	Calibration range (mg/L)	Linear range equation*	R2
Campesterol	0.05-50	y= 0.431x - 0.00731	0.9976
Stigmasterol	0.1-50	y= 0.734x - 0.0157	0.9978
Sitosterol	0.05-50	y= 0.409x - 0.00511	0.9976
Lanosterol	0.05-50	y= 0.809x - 0.0103	0.9982
Desmosterol	0.05-50	y= 1.08x - 0.017	0.9991

* assessed based on n=3 calibration curves.

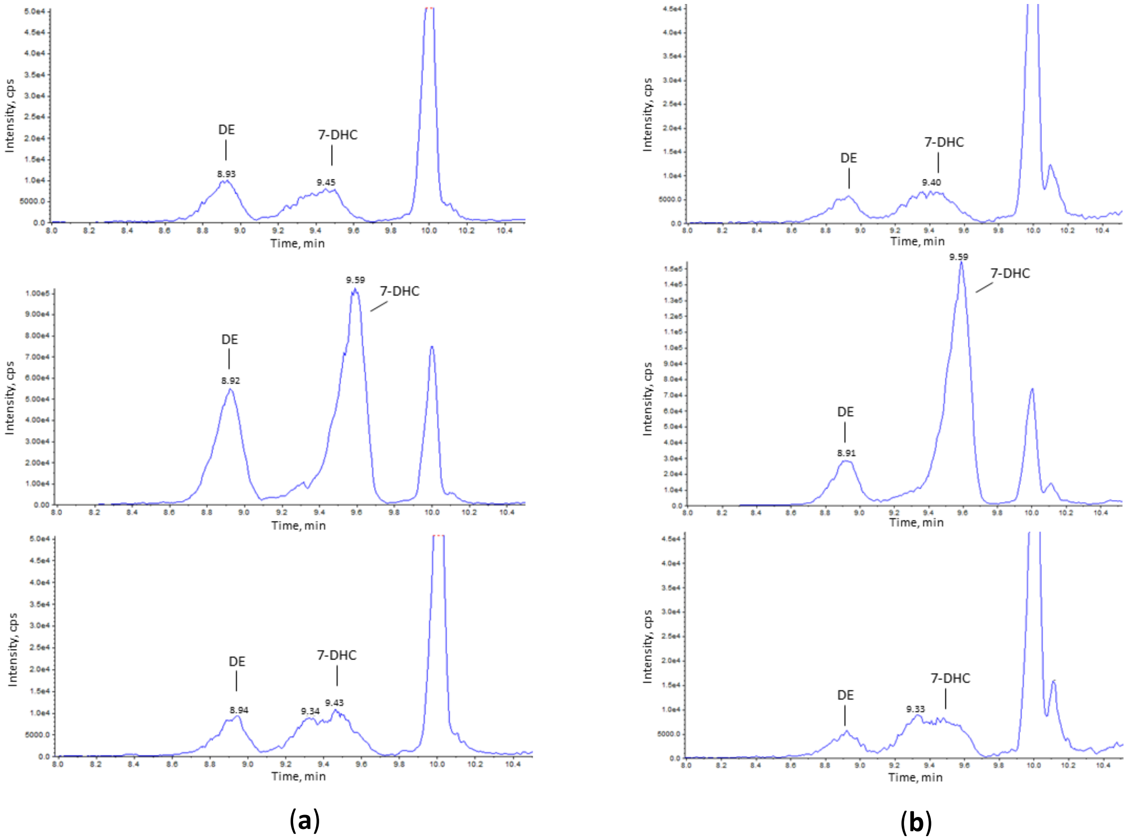

Figure S2. Representative chromatograms of DE and 7-DHC. (a) Plasma chromatogram when DE precursor/transition is selected (m/z: 367.4/161.2). (b) Plasma chromatogram when 7-DHC precursor/transition is selected (m/z: 367.3/91). Upper graphs- pooled plasma sample (QC); middle graphs- DE and 7-DHC spiked pooled plasma; lower graphs- pooled plasma before spikes.

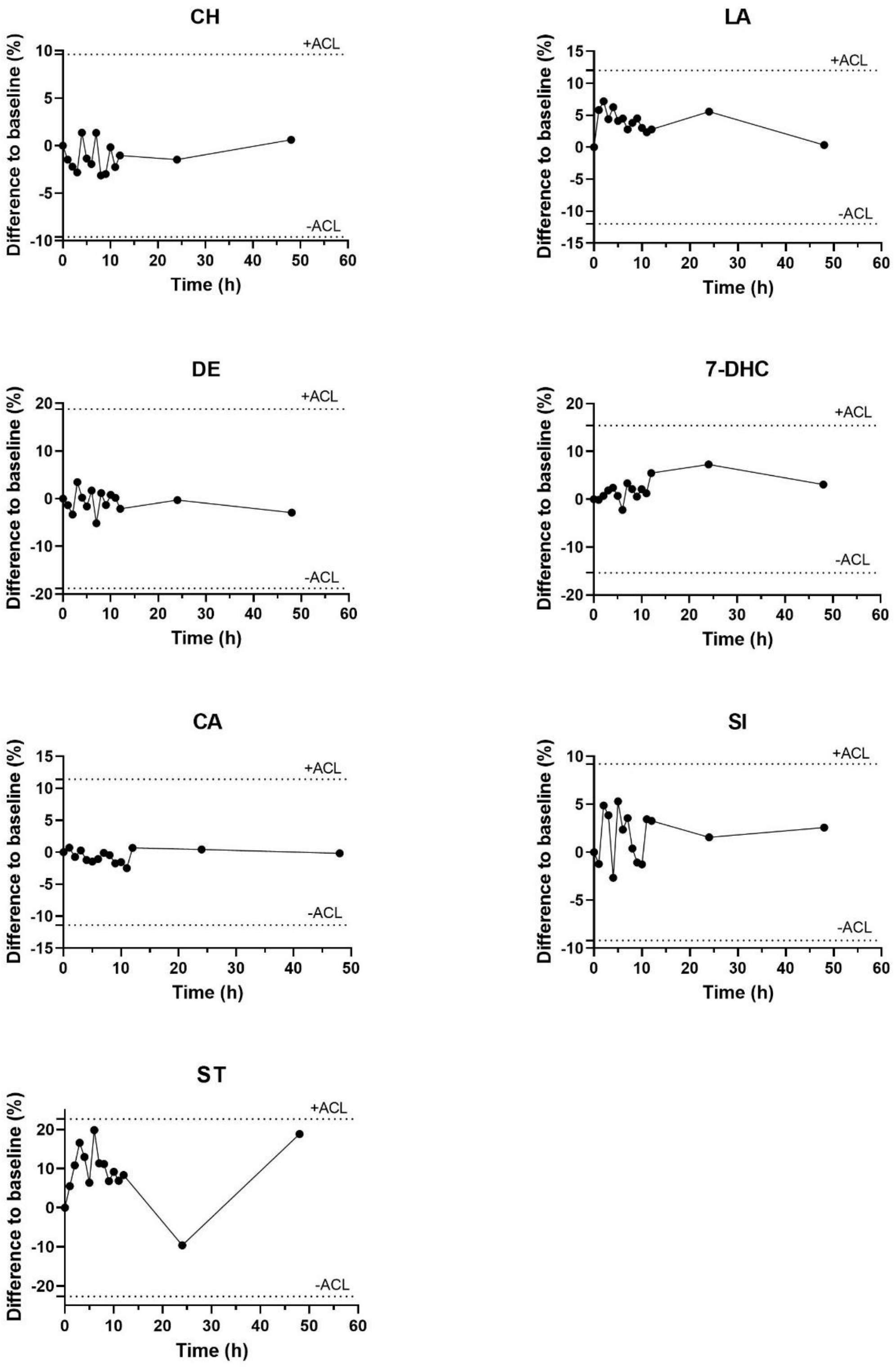

Figure S3. Stability of sterols in EDTA-plasma at 10 °C over 48 hours. Stability was assessed in the plasma pool samples (n=3) every hour for 12 hours, after 24 and after 48 hours. Result for every time point is presented as a percentage difference to the mean baseline value (T=0). Acceptable change limit (ACL) was figured as dashed line.

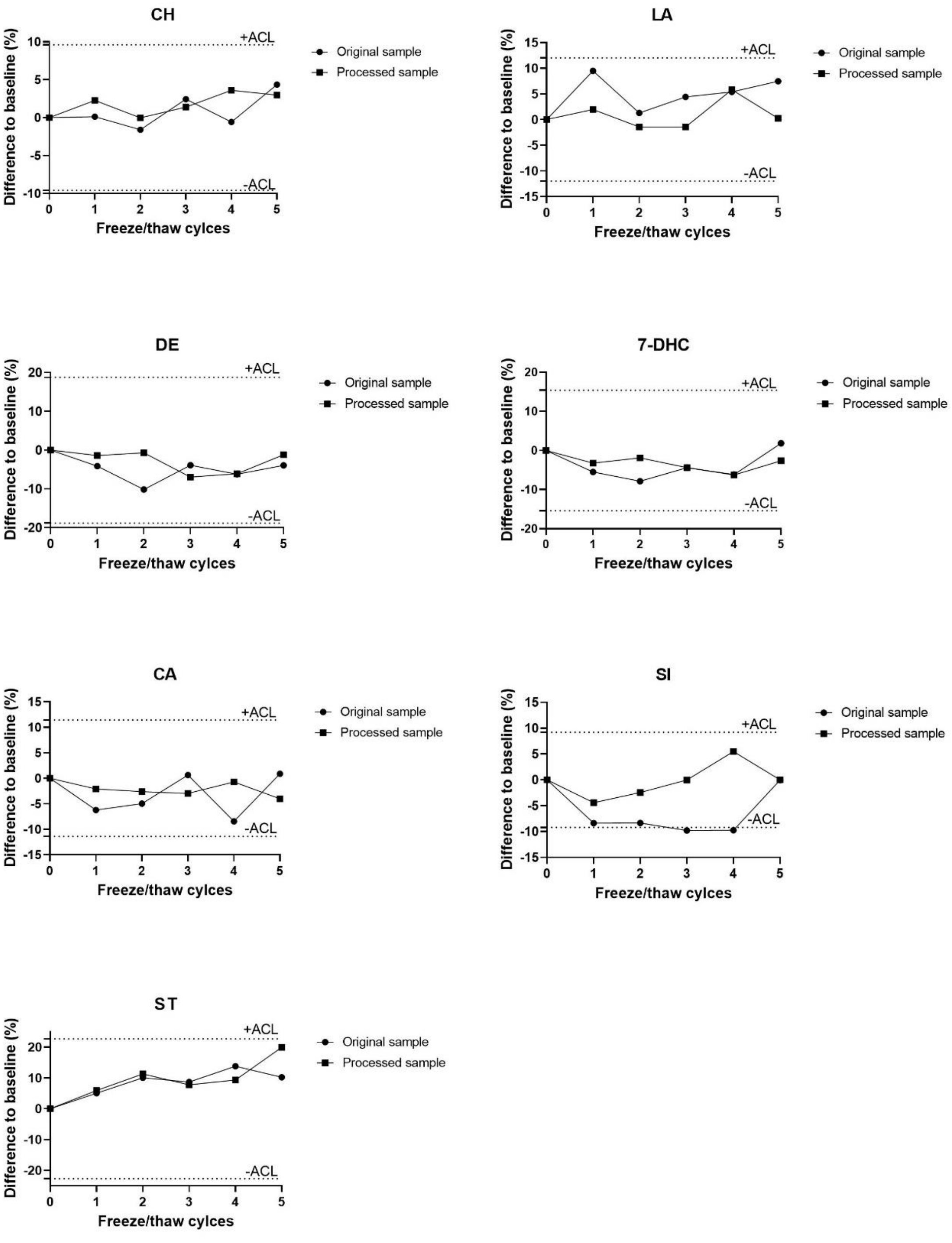

Figure S4. Stability of sterols in EDTA-plasma (native and processed) during repeated freeze and thaw cycles. Once frozen and thawed original plasma pool samples (n=3, T=0) and processed samples (n=3) were subjected for additional 5 cycles of freeze and thaw. Result for every time point is presented as a percentage difference to the mean (n=3) baseline value (T=0). Acceptable change limit is figured as dashed line.

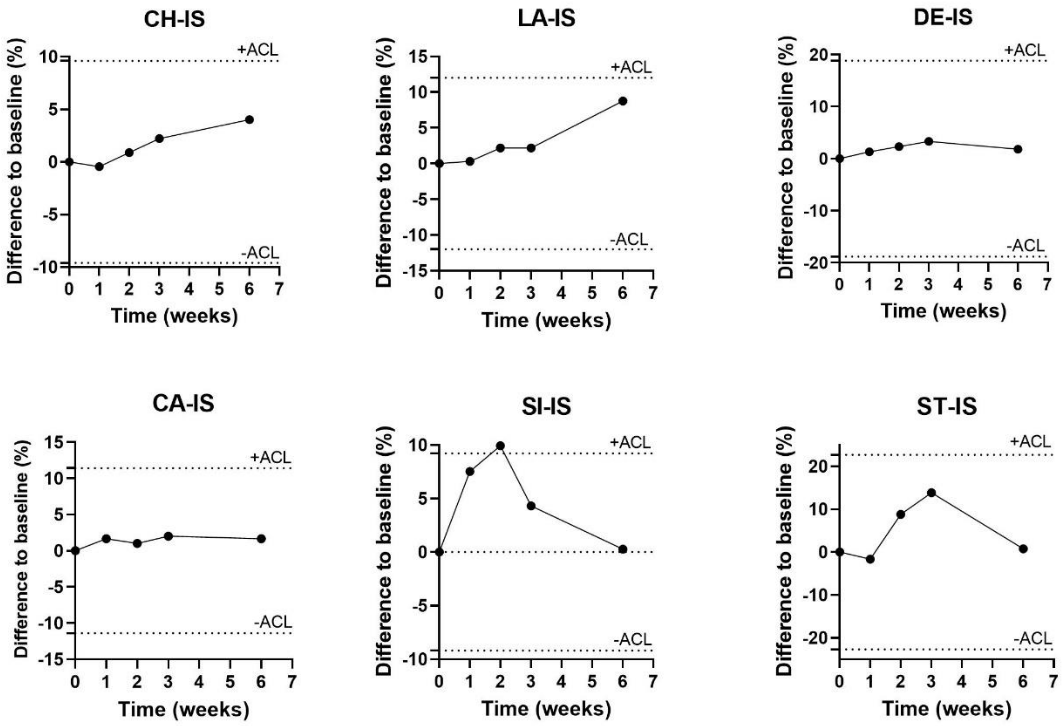

Figure S5. Internal standard stability stored at -50 °C over time. Stability of the internal standard working solution was assessed with a plasma pool sample (n=1) prepared after one, two, three and six weeks after initial preparation (T=0). Result for every time point is presented as a percentage difference to the mean (n=3) baseline value (T=0). Acceptable change limit (ACL) is figured as dashed line.

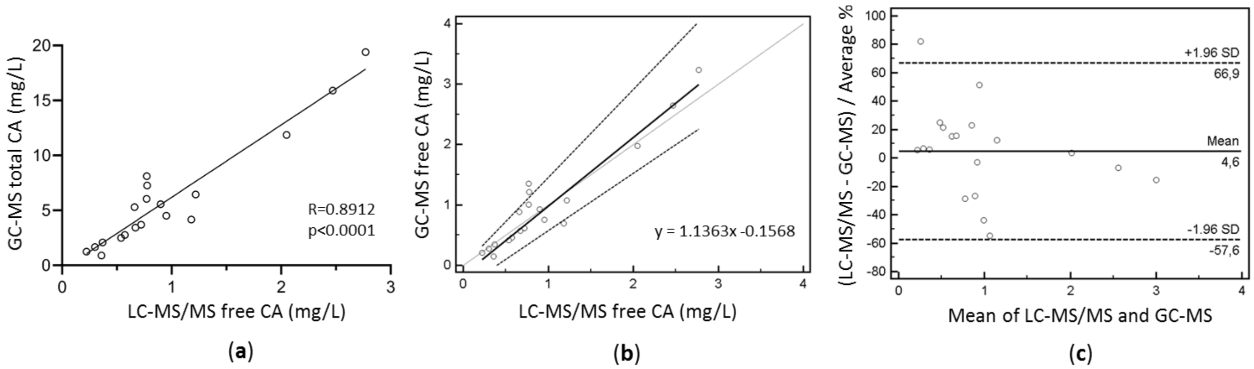

Figure S6. Method comparison of the established sterol LC-MS/MS assay with the GC-MS assay. Comparison was performed using human samples (n=19) and analyzing CA with both methods; (**a**) Spearman correlation between total (GC-MS) and free CA (LC-MS/MS) levels; (**b**) Passing-Bablok regression between estimated free CA with GC-MS and free CA by LC-MS/MS (95% CI of the slope 0.9480-1.4564 and intercept -0.3728-(-0.001466)); (**c**) Bland-Altman plot of estimated mean difference and standard deviations (SD) of CA levels between the two methods (total CA concentration).

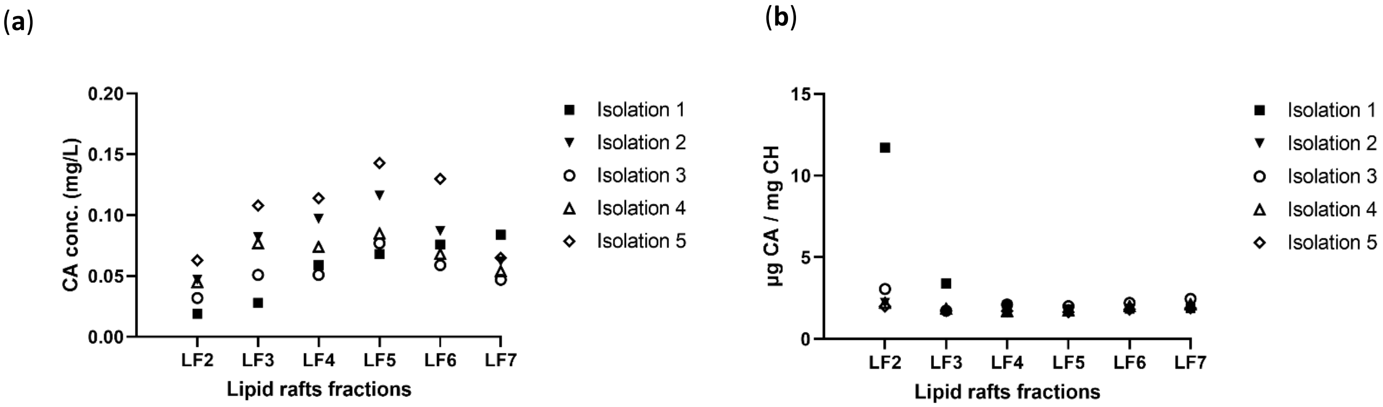

Figure S7. Campesterol quantitative abundance in membrane microdomain fractions. (a) Campesterol (CA) concentrations in lipid rafts fractions (LF) 2-7 in five consecutive isolation, expressed as mg/L; (b) CA concentration in the same fractions normalized by cholesterol (CH) levels, expressed as µg CA/mg CH.

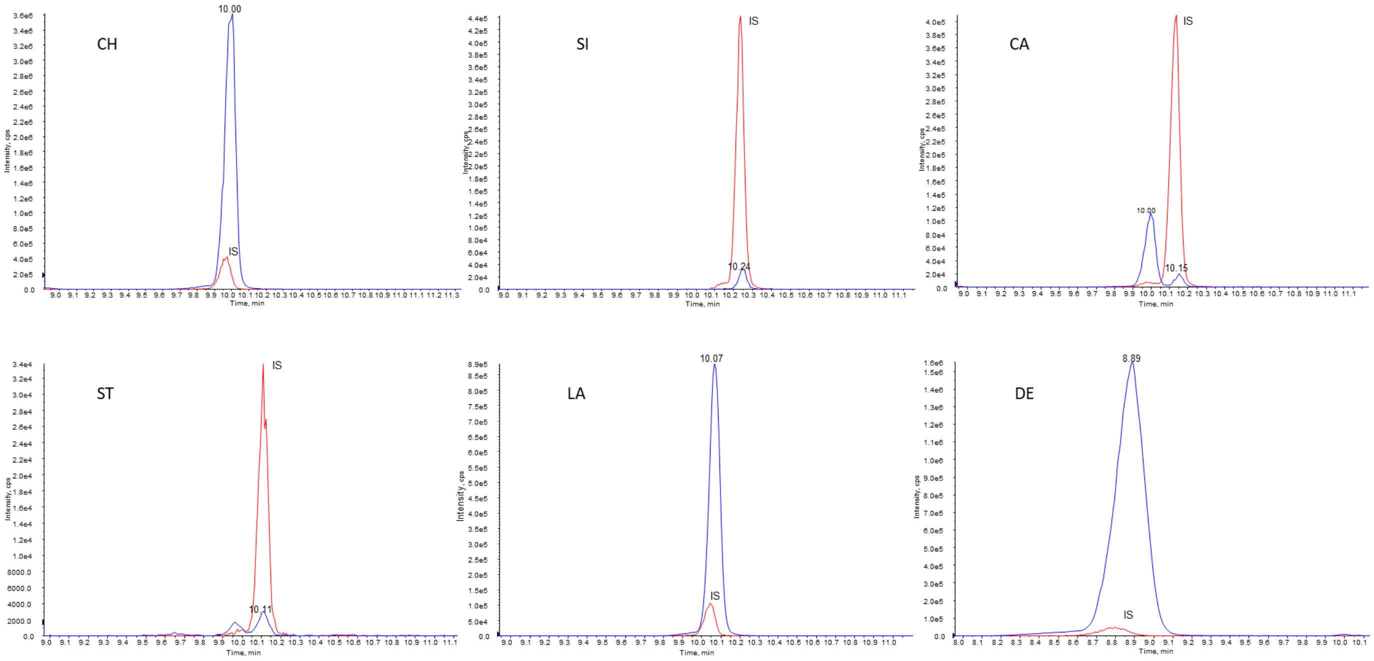

Figure S8. Representative chromatograms of sterols in fraction 5. Chromatograms of sterols and its internal standards are given for cholesterol (CH), sitosterol (SI), campesterol (CA), stigmasterol (ST), lanosterol (LA) and desmosterol (DE).
